# A novel score to estimate thrombus burden and predict intracranial hypertension in cerebral venous sinus thrombosis

**DOI:** 10.1186/s10194-023-01562-9

**Published:** 2023-03-17

**Authors:** Zhongao Wang, Chaitu Dandu, Yibing Guo, Meini Gao, Duo Lan, Liqun Pan, Da Zhou, Yuchuan Ding, Xunming Ji, Ran Meng

**Affiliations:** 1 Department of Neurology, National Center for Neurological Disorders, Xuanwu Hospital, Capital Medical University, Beijing, 100053 China; 2 Advanced Center of Stroke, Beijing Institute for Brain Disorders, Beijing, 100053 China; 3 Department of Neurosurgery, Wayne State University School of Medicine, Detroit, Michigan 48201 USA; 4 Department of Neurosurgery, Xuanwu Hospital, Capital Medical University, Beijing, 100053 China

**Keywords:** Cerebral venous sinus thrombosis, Scoring method, Thrombus burden, Intracranial pressure, Magnetic resonance black-blood thrombus imaging

## Abstract

**Background:**

Current methods to evaluate the severity of cerebral venous sinus thrombosis (CVST) lack patient-specific indexes. Herein, a novel scoring method was investigated to estimate the thrombus burden and the intracranial pressure (ICP) of CVST.

**Methods:**

In this retrospective study from January 2019 through December 2021, we consecutively enrolled patients with a first-time confirmed diagnosis of CVST by contrast-enhanced magnetic resonance venography (CE-MRV) or computed tomography venography (CTV). In these patients, a comprehensive CVST-Score was established using magnetic resonance black-blood thrombus imaging (MRBTI) to estimate the thrombus burden semi-quantitatively. The relationship between CVST-Score and ICP was explored to assess the potential of using the CVST-score to evaluate ICP noninvasively and dynamically.

**Results:**

A total of 87 patients were included in the final analysis. The CVST-Scores in different ICP subgroups were as follows: 4.29±2.87 in ICP<250mmH_2_O subgroup, 11.36±3.86 in ICP =250-330mmH_2_O subgroup and 14.99±3.15 in ICP>330mmH_2_O subgroup, respectively (*p*<0.001). For patients with ICP ≤330mmH_2_O, the CVST-Score was linearly and positively correlated with ICP (*R*^2^=0.53). The receiver operating characteristic (ROC) curves showed the optimal CVST-Score cut-off values to predict ICP ≥250mmH_2_O and >330mmH_2_O were 7.15 and 11.62, respectively (*P*<0.001). Multivariate analysis indicated CVST-Score as an independent predictor of ICP ≥250mmH_2_O (odds ratio, 2.15; 95% confidence interval, 1.49-3.10; *p*<0.001).

**Conclusions:**

A simple and noninvasive CVST-Score can rapidly estimate the thrombus burden and predict the severity of intracranial hypertension in patients with CVST. The CVST-Score can aid in evaluating therapeutic responses and avoiding unnecessary invasive procedures at long-term follow-up.

**Graphical Abstract:**

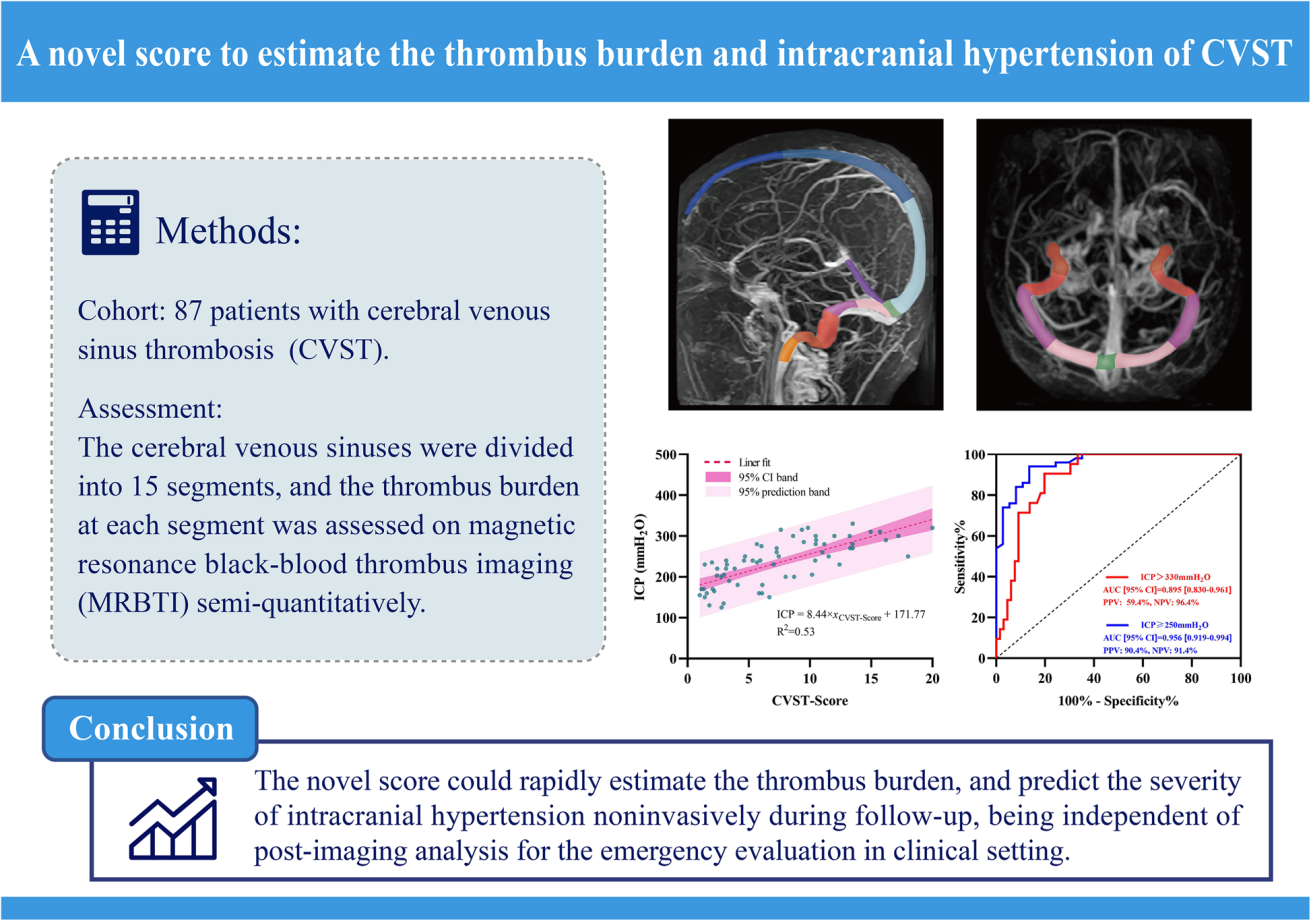

**Supplementary Information:**

The online version contains supplementary material available at 10.1186/s10194-023-01562-9.

## Introduction

It is commonly known that patients with extensive cerebral venous sinus thrombosis (CVST) are prone to intracranial hypertension and visual impairment. In addition, severe and persistent cerebral venous outflow obstruction with subsequent intracranial hypertension may contribute to brain damage from CVST [1–4]. Although most patients with CVST were functionally independent as measured with the modified rankin scale (mRS) during long-term follow-up [5], a large proportion of them still had a series of persistent neurologic sequelae such as severe residual headaches, neuropsychological problems, and long-term visual disorder in addition to the focal neurological deficits by cerebral infarction. These symptoms may be related to the substantial residual thrombus burden of CVST and, more importantly, hinder a patient’s ability to return to previous work [6–10].

Therefore, constantly monitoring the thrombus burden and intracranial pressure (ICP) would contribute to estimating the severity and therapeutic responses of CVST. Previous methods for calculating thrombus burden of CVST only described the location of thrombi and roughly assessed thrombus burden at each sinus with a binary scoring rule. Due to the limited imaging techniques present during the development of these methods, some important features may be ignored [1, 3, 11, 12]. In addition, repeated lumbar puncture for ICP monitoring is risky and unethical, especially in patients with severe intracranial hypertension and on anticoagulant therapy [13]. Hence, a noninvasive method to evaluate the thrombus burden and ICP in CVST is required to monitor disease status and guide further therapy. Unfortunately, to our knowledge, no scoring method has been established to simultaneously perform both functions of evaluating thrombus burden and measuring ICP.

Magnetic resonance black-blood thrombus imaging (MRBTI), a T1-weighted imaging method, has been used more frequently to diagnose CVST. It can visualize the thrombus directly by effectively suppressing blood signals and has the potential to quantify thrombus volume [14–16]. However, thrombus volume quantification on MRBTI maps requires cumbersome and time-consuming post-imaging analysis, which is unsuitable for emergency evaluations in the clinical setting. In light of this, we aim to propose a comprehensive and semi-quantitative scoring method based on MRBTI, which considers multiple factors: the segmentation of thrombi, the thrombus burden at each segment, and the dominance of transverse sinus. More importantly, these factors do not need post-imaging analysis, allowing this scoring method to be used in emergencies. Additionally, we would investigate the relationship between the score and ICP to explore its potential to monitor ICP noninvasively during regular follow-up visits. We would also compare the capacity of the novel CVST-Score for diagnosing intracranial hypertension with two other conventional scoring methods commonly used in recent clinical research studies.

## Methods

We retrospectively analyzed consecutive patients with a first-time diagnosis of CVST in the Neurology department at Xuanwu Hospital, Capital Medical University, from January 2019 to December 2021. The study was approved by the Institutional Ethic Committee of Xuanwu Hospital, Capital Medical University in accordance with the Declaration of Helsinki. Written informed consent was obtained from the patients who also took part in our ongoing cohort studies by face-to-face interviews and mails. The Institutional Ethic Committee waived the informed consent requirement for patients who could not be contacted. Patients with CVST underwent lumbar puncture due to the suspicious symptoms of intracranial hypertension such as headache and visual impairment, or clinical signs of papilledema, when the contraindications were excluded. A lumbar puncture was performed within two days of admission. ICP was measured with a disposable sterile polyethylene manometer tube in the lumbar puncture kit with a measuring range from 50mmH_2_O to 330mmH_2_O without an extension tube. Before any invasive procedure, written informed consent was obtained from the patient. MRBTI was performed within three days of admission per our institution’s standardized clinical protocol to confirm the thrombus burden for further therapy guidance.

The inclusion criteria included: (a) a first-time diagnosis of CVST by contrast-enhanced magnetic resonance venography (CE-MRV) or computed tomography venography (CTV) in our institution; (b) complete the MRBTI scan within three days after admission; (c) indications for lumbar puncture such as symptoms of intracranial hypertension or clinical signs of papilledema; (d) no restriction regarding gender or age. The exclusion criteria were as follows: (a) failure to get cerebrospinal fluid (CSF) opening pressure, with contraindications to lumbar puncture or refusing lumbar puncture within two days after admission; (b) intracranial lesions possibly affecting the ICP such as neoplasms; (c) non-thrombotic cerebral venous sinus stenosis (CVSS) such as giant arachnoid granulations; (d) cardiac dysfunction or superior vena cava syndrome, which could restrict cerebral venous outflow; (e) definite intra- or extracranial arterial stenosis; (f) comorbid austere diseases; (g) severe renal or liver dysfunction or malignant tumors; (h) insufficient clinical or imaging data.

### Assessment

**Fig. 1:**
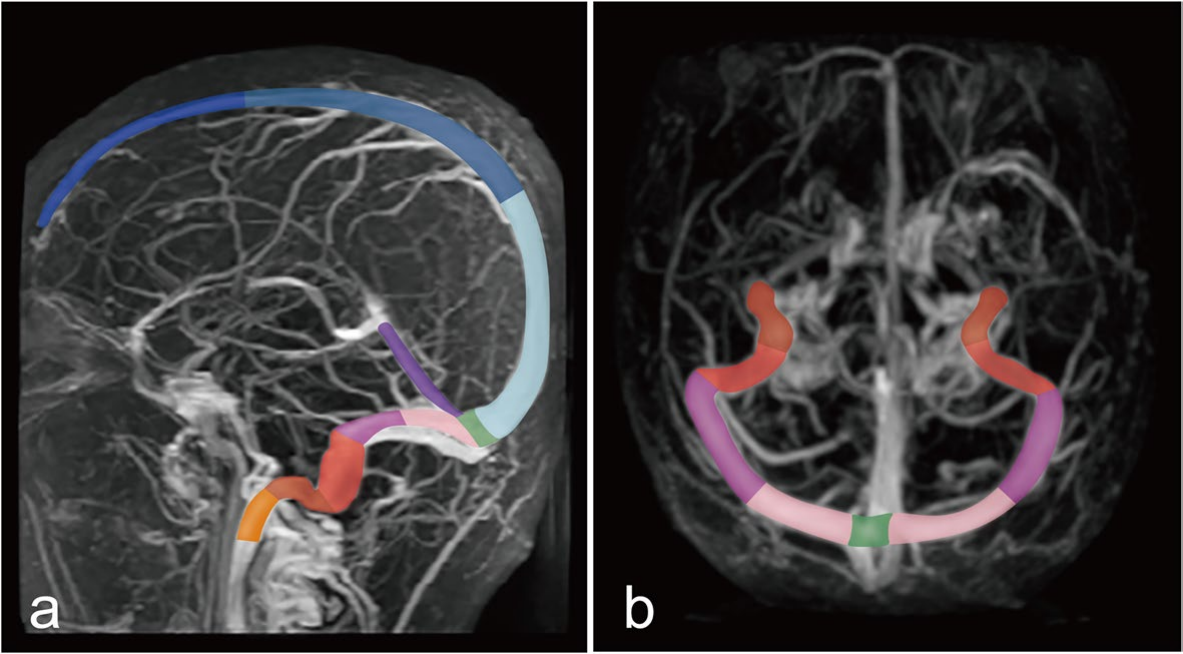
Segmentation of cerebral venous sinuses in the novel CVST-Score. The superior sagittal sinus and the unilateral transverse sinus were equally divided into three segments (dark blue, medium blue and light blue) and two segments (light pink and dark pink), respectively. The unilateral sigmoid sinus was divided into two segments at the bend: the vertical and horizontal segments (light red and dark red). The straight sinus (purple), torcular herophili (green) and unilateral internal jugular vein (orange-yellow) were all just counted as one segment (**a**. sagittal, **b**. axial)

We designed a novel method to calculate the thrombus burden of CVST, named the CVST-Score. Firstly, we divided the cerebral venous sinuses into 15 segments: the superior sagittal sinus and the unilateral transverse sinus were equally divided into three segments and two segments, respectively. The unilateral sigmoid sinus was divided into two segments at the bend: the vertical and horizontal segments. The straight sinus, torcular herophili and unilateral internal jugular vein were all just counted as one segment each [3] (Fig. 1). The scoring rules for each segment were as follows: no thrombus was counted as zero points, thrombus filling ≤50% of the segment as one point, thrombus filling >50% but <100% of the segment as two points, and thrombus filling 100% of the segment as three points. Moreover, considering the common unilateral dominance of transverse sinus, we manually measured the maximal cross-sectional area of bilateral transverse sinuses to calculate the weighted drainage of each side.

Calculation formulas for the novel method were summarized in three steps as follows: The first step was calculating the weighted drainage of the left transverse sinus: W_L_= CS_L_ / (CS_L_+CS_R_), and that of the right side: W_R_= CS_R_ / (CS_L_+CS_R_). (Abbreviations: CS_L_, the maximal cross-sectional area of left transverse sinus; CS_R_, the maximal cross-sectional area of right transverse sinus) The second step was calculating the weighted score on the left side of cerebral venous sinuses: A_L_= (R_L_+ I_L_+J_L_)×W_L_, then that on the right side: A_R_= (R_R_+ I_R_+J_R_)×W_R_. (Abbreviations: the capital letters “R”, “I” and “J” represented the total thrombus scores of “transverse sinus”, “sigmoid sinus” and “internal jugular vein” at the ipsilateral side, respectively; the subscript “L” and “R” represented the “left” and “right” side; the “W_L_” and “W_R_” were as mentioned above) The final step was calculating the total thrombosis score: CVST-Score = S + T + O + A_L_ + A_R._ (Abbreviations: the capital letters “S”, “T” and “O” represented the “total score of superior sagittal sinus”, “score of straight sinus” and “score of torcular herophili”; the “A_L_” and “A_R_” were as mentioned above) The minimum and maximum total scores were 0 and 30, respectively. Higher scores represented higher thrombus burden. Additionally, a form was designed for recording data and calculating the CVST-Score (Details in Table S1).

The diagnostic capacity of the novel CVST-Score was assessed against two conventional scoring methods commonly used in clinical research studies, which remained the mainstream to report the thrombus burden of CVST for each patient [1, 3, 11, 12, 17–23]. Though CVST guidelines do not endorse any methods for assessing thrombus burden, these two methods are widely accepted as the golden standards for assessing the thrombus volume [9]. The scoring rule for the first conventional method is simply composed of the location and number of involved sinuses, which was called the cerebral venous extent score (CVES) in this study according to the nomenclature [3, 12, 19–22]. The cerebral venous sinuses were divided into seven segments: the superior sagittal sinus, straight sinus, torcular herophili and unilateral internal jugular vein were each counted as one segment. In addition, the ipsilateral transverse sinus and sigmoid sinus were combined and called the lateral sinus as one segment [12, 20]. A segment received one point if it contained thrombus and zero points if no thrombus was present. The final score is calculated by summing all the segment scores [3, 12, 20].

Similarly, the second conventional scoring method, the cerebral venous occlusion score (CVOS), was based on whether the venous sinus was fully occluded [1, 11, 17, 18, 23]. The cerebral venous sinuses were segmented the same way as the sinuses in the CVES method. However, in the CVOS scoring method, the segment only received a point if it was fully occluded; segments with partial thrombus or no thrombus received zero points. The final score is also calculated by taking the sum of all segment scores [1, 11, 18].

### MRBTI protocol and image evaluation

MRBTI data was acquired using a 3.0T MRI system (MAGNETOM Verio, Siemens Healthcare, Erlangen, Germany) with a standard 32-channel head coil for signal reception. The specific imaging parameters were described in our center’s previous work [14], including oblique coronal single-slab coverage, repetition time (TR) = 800 milli-seconds, echo time (TE) = 22 milli-seconds, matrix = 198×192, FOV = 160×200 mm^2^, slice thickness = 0.6-1.0 mm, slices = 100-200, and scan time = 6-8 minutes.

All MRBTI images were retrospectively reviewed in a randomized, blinded fashion by two independent readers with seven years (D.Z.) and ten years (R.M.) of experience in diagnosing CVST, respectively. The readers were trained to calculate the CVST-Score on 20 patients independent from the study population. The training set was collected from patients who did not meet the inclusion criteria or were excluded from the study. The readers were blinded to general information, clinical data, and conventional images. In addition, source images and multiplanar reconstruction images were used for more precise thrombus evaluation. A third reader with 11 years of experience in reading (XM.J.) resolved any dispute between the two readers. The readers (R.M. and XM.J.), who contributed to the conception and design of the score, are chief physicians in the Center for Intracranial Hypertension & Cerebral Venous Disease of China’s National Health Commission in Xuanwu Hospital and have seen patients from all over China. They have accumulated more than ten years of experience in diagnosing and treating CVST, and are also the chief neurologists for a series of clinical studies on CVST.

### Statistical analysis

Continuous variables were expressed as mean ± standard deviation (SD) for normally distributed variables; otherwise, they were presented as median (IQR). Categorical variables were depicted as numbers (percentages). The normality of the distribution of continuous variables was visually assessed using histograms and probability distribution plots. Intra-class correlation coefficient (ICC) was calculated to assess inter-reader reliability. The degree of ICC was classified as follows: below 0.50: poor; 0.50–0.75: moderate; 0.75–0.90: good; above 0.90: excellent. Once the scores were deemed excellent, both readers’ results were averaged. Differences in thrombus scores among multiple ICP subgroups were tested using one-way ANOVA followed by post hoc Tukey comparison for normally distributed variables and the Kruskal-Wallis test for non–normally distributed variables. In patients with ICP≤330mmH_2_O, the correlation between CVST-Score and ICP was evaluated by univariate linear regression analysis. The receiver operating characteristic (ROC) curves were constructed to calculate the areas under the curves (AUC). The maximum value of the Youden index was used to identify the optimal cut-off values of the CVST-Score and the conventional scoring methods to predict ICP. Multivariable binary logistic regression analysis served to determine independent factors associated with ICP. Due to the differences in demographics and imaging findings among the ICP subgroups, we considered sex, age, body mass index (BMI), onset-to-door time, and cerebral infarction as potential confounding factors when predicting ICP with the novel CVST-Score. The onset-to-door time was defined as the time from symptom onset to hospital admission in our center. Univariate odds ratios (ORs) and 95% confidence intervals (CIs) were calculated for each variable. The Box-Tidwell method was utilized to check the linearity of continuous variables. Multicollinearity was tested by a variation inflation factor (<10 considered acceptable). Then, the retained variables with *P* < 0.10 were entered into the multivariate analysis, and further adjusted for age, cerebral infarction, and BMI. A two-sided *P* < 0.05 was considered statistically significant. All statistical analyses were conducted using SPSS Version 21.0 (IBM Corp., Armonk, NY, USA).

## Results

### Clinical presentation

**Fig. 2:**
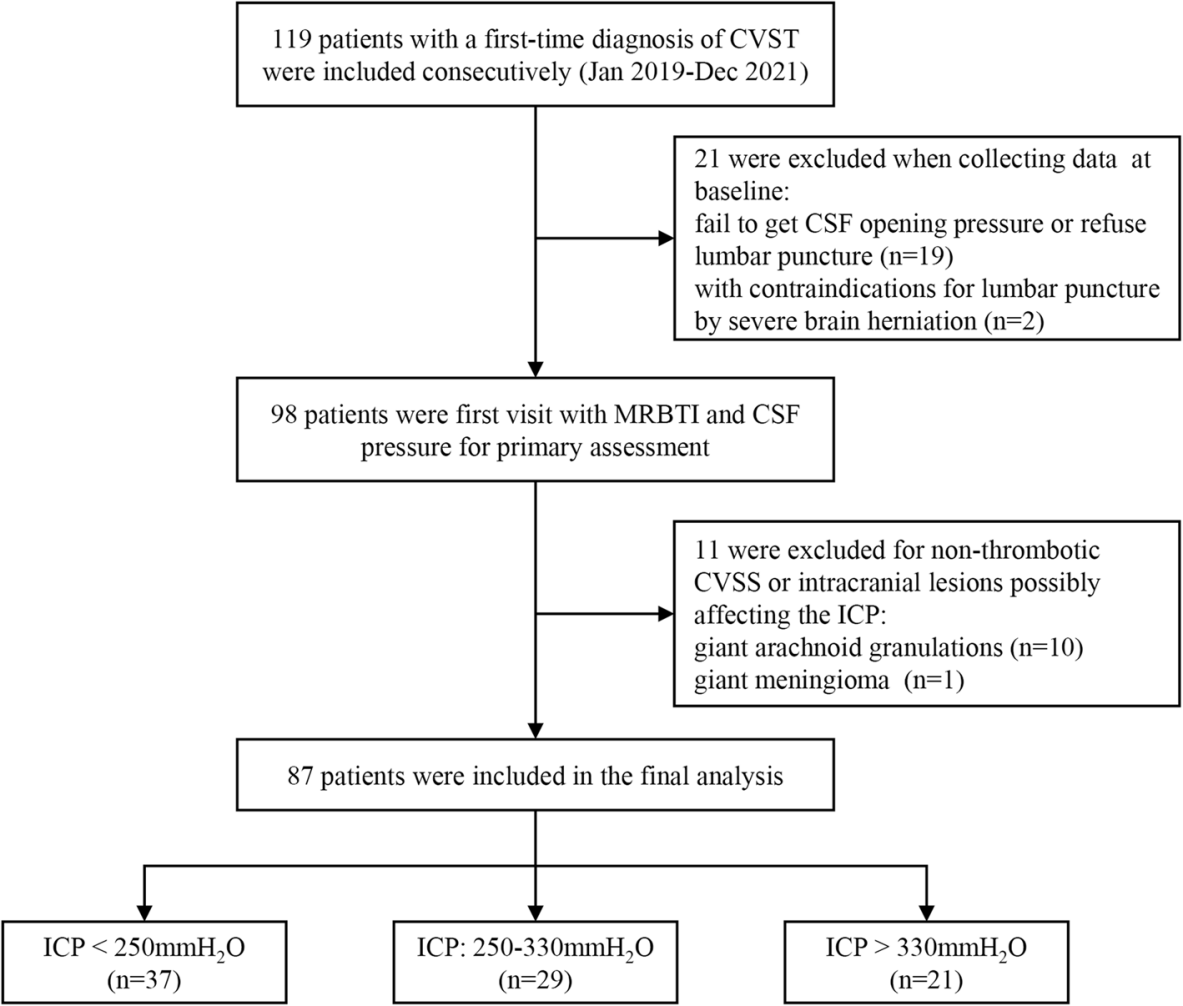
Patient flow chart

A total of 119 eligible patients with a first-time diagnosis of CVST in our institution were enrolled consecutively. After initial assessment, 32 patients were excluded. Reasons for exclusion include 19 patients failing to get CSF opening pressure or refusing lumbar puncture after admission; two patients had contraindications to lumbar puncture due to severe brain herniation including cerebral ventricular compression and a midline shift; ten patients had non-thrombotic CVSS due to giant arachnoid granulations; and one patient had giant meningioma affecting the ICP. A flow chart of the detailed enrollment process is shown in Fig. 2. After exclusions, 87 patients (50 males and 37 females) were included in the final analysis. The average age was 39.03±13.08 years (range, 15-66 years). The median onset-to-door time was 3.50 (IQR, 1-11) months. The average BMI was 25.19±3.84 kg/m^2^.

Patients were stratified in groups according to their ICP; the numbers in each subgroup were as below: 37 (42.5%) in ICP<250mmH_2_O subgroup, 29 (33.3%) in ICP being 250-330mmH_2_O subgroup, and 21 (24.1%) in ICP>330mmH_2_O subgroup. Headache (88.5%) was the most common symptom, followed by nausea or vomiting (63.2%), visual impairment (33.3%), tinnitus (26.4%), dizziness (26.4%), and double vision (14.9%). Among the 87 patients, 47 (54.0%) had papilledema, and 33 (37.9%) had visual field defects. Risk factors present among patients with CVST included prothrombotic conditions (42.5%), oral contraceptives (8.0%), pregnancy and puerperium (2.3%), systemic diseases (11.5%), hematologic disorders (9.2%), and local infection (5.7%). A complete list of clinical presentation is displayed in Table 1.

**Table 1:** Clinical features of CVST

**Clinical features**	
**Demographics**	
No. of patients	87
Gender (male/female)	50/37
Age (years) (Mean±SD)	39.03±13.08
Onset-to-door time (months) (Median, IQR)	3.5 (IQR, 1-11)
BMI (kg/m^2^)	25.19±3.84
**Symptoms and signs (No., %) *n*=87**	
Headache	77 (88.5)
Nausea or vomiting	55 (63.2)
Visual impairment	29 (33.3)
Tinnitus	23 (26.4)
Dizziness	23 (26.4)
Double vision	13 (14.9)
Papilledema	47 (54.0)
Visual field defect	33 (37.9)
**Risk factors (No., %) *n*=87**	
Prothrombotic conditions^a^	37 (42.5)
Oral contraceptives	7 (8.0)
Pregnancy and puerperium	2 (2.3)
Systemic diseases^b^	10 (11.5)
Hematologic disorders^c^	8 (9.2)
Local infection	5 (5.7)
Drugs^d^	3 (3.4)
Malignancy	2 (2.3)
Surgery/trauma	1 (1.1)
None identified	12 (13.8)

*Abbreviations*: *CVST* Cerebral venous sinus thrombosis, *IQR* Inter quartile range, *SD* Standard deviation, *BMI* Body mass index

^a^Prothrombotic conditions included antithrombin III deficiency, protein C/S deficiency, antiphospholipid antibody, hyperhomocysteinemia, and nephrotic syndrome

^b^Systemic diseases included thyroid disease and Behçet disease

^c^Hematologic disorders included polycythemia, thrombocythemia, and anemia

^d^Drugs included steroids and intravenous immunoglobulin

### Imaging presentations

Among the 87 patients, 17 (19.5%) had concomitant cerebral infarction by CVST but did not have imaging findings suggestive of severe brain herniation, such as cerebral ventricular compression or midline shifts. The anatomic sites of the thrombi distribution among patients were as follows: superior sagittal sinus (60.9%), straight sinus (26.4%), torcular herophili (21.3%), right transverse sinus (80.5%), right sigmoid sinus (63.2%), right jugular vein (58.0%), left transverse sinus (83.3%), left sigmoid sinus (47.1%), and left jugular vein (40.8%). The time from admission to MRBTI scanning was 56.29±7.22 hours. Each reader took an average of 3.62±0.68 minutes to calculate the CVST-Score.

### Correlation between CVST-Score and ICP

**Fig. 3:**
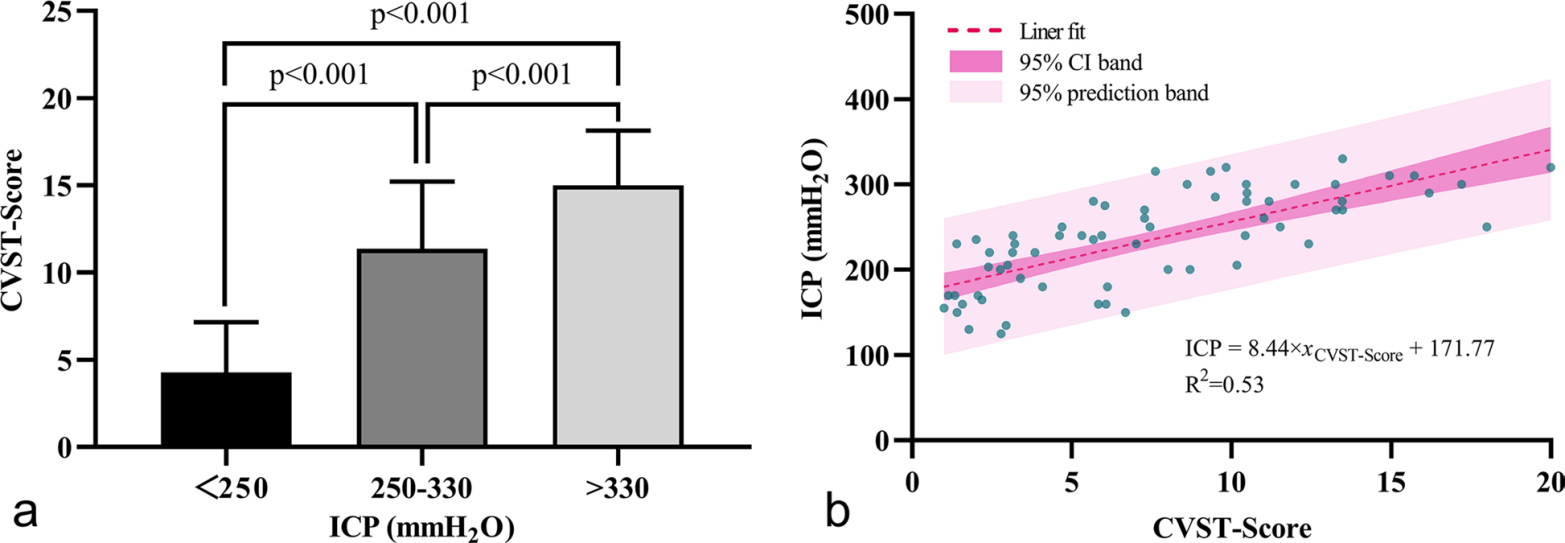
The correlation between the CVST-Score and ICP. CVST-Score in patients with ICP <250mmH_2_O, 250-330mmH_2_O and >330mmH_2_O (panel **a**). Scatterplot of CVST-Score vs. ICP (panel **b**), and the line of best fit (95% CI band and 95% prediction band) for CVST-Score predicting ICP in patients with ICP≤330 mmH_2_O. For every unit increase in CVST-Score, the associated ICP increases by 8.44 mmH_2_O. Abbreviations: CVST, cerebral venous sinus thrombosis; ICP, intracranial pressure; CI, confidence interval.

There was high reliability between the two readers’ scores. The ICC for the observers was 0.99 (95% CI 0.98 to 0.99) with the novel CVST-Score, 0.93 (95% CI 0.90 to 0.96) with the CVES method, and 0.91 (95% CI 0.86 to 0.94) with the CVOS method. ICP >330mmH_2_O could not be obtained due to technical limitations in the measuring range of the CSF opening-pressure manometer. Therefore, the patients were divided into three subgroups according to ICP range: <250mmH_2_O, 250-330mmH_2_O, and >330mmH_2_O. The CVST-Score of each subgroup was 4.29±2.87, 11.36±3.86, and 14.99±3.15, respectively (*p*<0.001). Details are displayed in Fig. 3 (panel a). Correspondingly, the score of each subgroup was 2.72±0.92, 4.10±1.27, and 4.98±1.07 with the CVES method (*p*<0.001), and 0 (IQR, 0-0.5), 1 (IQR, 0-1.25), and 2 (IQR, 0-2.25) with the CVOS method (*p*<0.001). Details are given in Fig. S1 (panel a) and Fig. S2 (panel a). For patients with ICP ≤330mmH_2_O, the CVST-Score was linearly and positively correlated with ICP. The univariate linear regression analysis was as follows: *y*_ICP_ = 8.44×*x*_CVST-Score_ + 171.77 (R^2^=0.53, *p*<0.001). A scatterplot of CVST-Score vs. ICP is shown in Fig. 3 (panel b).

**Fig. 4:**
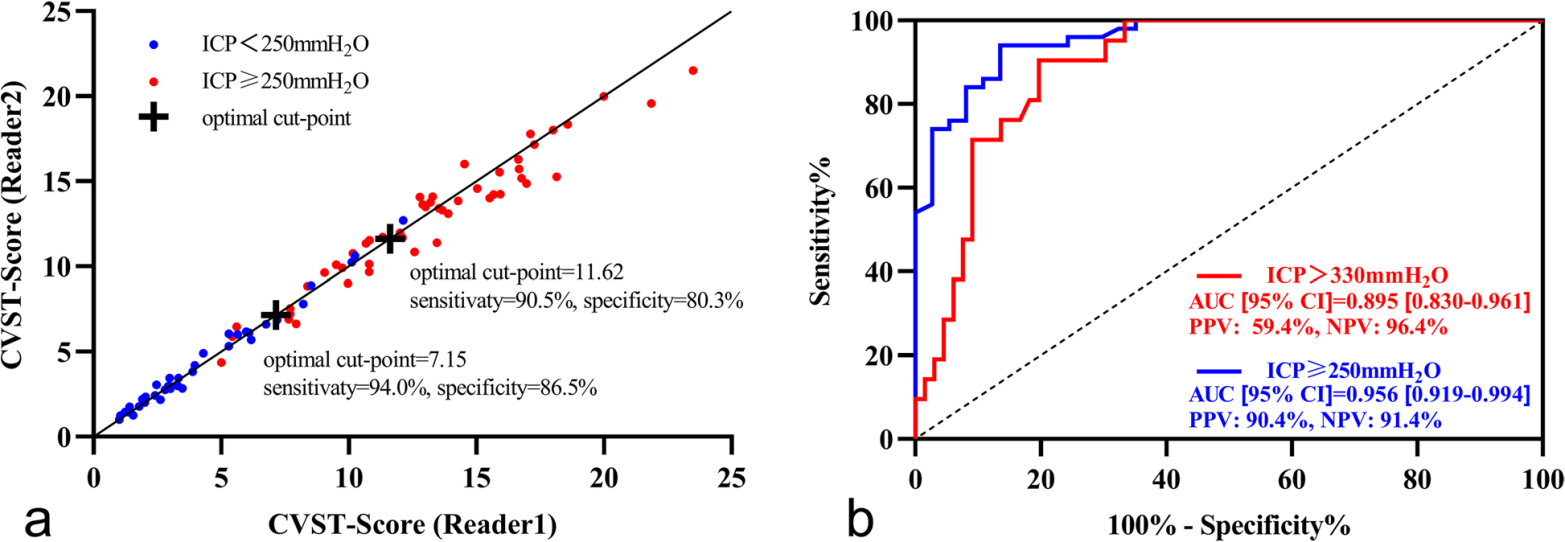
The inter-reader reliability and diagnostic accuracy of the CVST-Score. CVST-Score measured by each independent reader was plotted being separated by ICP≥250mmH_2_O or not, and the optimal cut-points for ICP diagnosis (≥250mmH_2_O or >330 mmH_2_O) were highlighted by a cross mark with corresponding sensitivity and specificity (panel **a**). The ROC curves for predicting ICP≥250mmH_2_O (blue) and ICP>330 mmH_2_O (red) are shown in panel **b**. Abbreviations: CVST, cerebral venous sinus thrombosis; ICP, intracranial pressure; ROC, receiver operating characteristic; PPV, positive predictive value; NPV, negative predictive value

We divided the patients into subgroups based on if their ICP was ≥250 mmH_2_O or >330 mmH_2_O. By plotting the ROC curves, the optimal cut-off values to predict ICP ≥250 mmH_2_O or >330 mmH_2_O were obtained with the novel CVST-Score, the CVES method and the CVOS method. The optimal threshold of CVST-Score to predict ICP ≥250 mmH_2_O was 7.15 (AUC = 0.956, 95% CI: 0.919-0.994, Youden index = 0.805, *p* < 0.001), and the sensitivity was 94.0%, specificity 86.5%, positive predictive value (PPV) 90.4%, and negative predictive value (NPV) 91.4%. The optimal threshold of CVST-Score to predict ICP >330 mmH_2_O was 11.62 (AUC = 0.895, 95% CI: 0.830-0.961, Youden index = 0.708, *p* < 0.001), and the sensitivity was 90.5%, specificity 80.3%, PPV 59.4%, and NPV 96.4%. Details are illustrated in Fig. 4 (panel b). Furthermore, when using the conventional CVES method, the optimal threshold to predict ICP ≥250 mmH_2_O was 3.25 (AUC = 0.863, 95% CI: 0.788-0.938, Youden index = 0.584, *p* < 0.001), and the sensitivity was 80.0%, specificity 78.4%, PPV 83.3%, and NPV 74.4%. The optimal threshold of the conventional CVES method to predict ICP >330 mmH_2_O was 3.75 (AUC = 0.834, 95% CI: 0.748-0.921, Youden index = 0.556, *p* < 0.001), and the sensitivity was 90.5%, specificity 65.2%, PPV 45.2%, and NPV 95.6%. Details are shown in Fig. S1 (panel b). Additionally, when assessing with the conventional CVOS method, the optimal threshold to predict ICP ≥250 mmH_2_O was 0.75 (AUC = 0.725, 95% CI: 0.620-0.830, Youden index = 0.384, *p* < 0.001), and the sensitivity was 60.0%, specificity 78.4%, PPV 78.9% and NPV 59.2%. The optimal threshold of the conventional CVOS method to predict ICP >330 mmH_2_O was 1.75 (AUC = 0.728, 95% CI: 0.586-0.871, Youden index = 0.465, p = 0.002), and the sensitivity was 57.1%, specificity 89.4%, PPV 63.2%, and NPV 86.8%. Details are reported in Fig. S2 (panel b). A summary of ROC analysis results is given in Table S2.

**Fig. 5:**
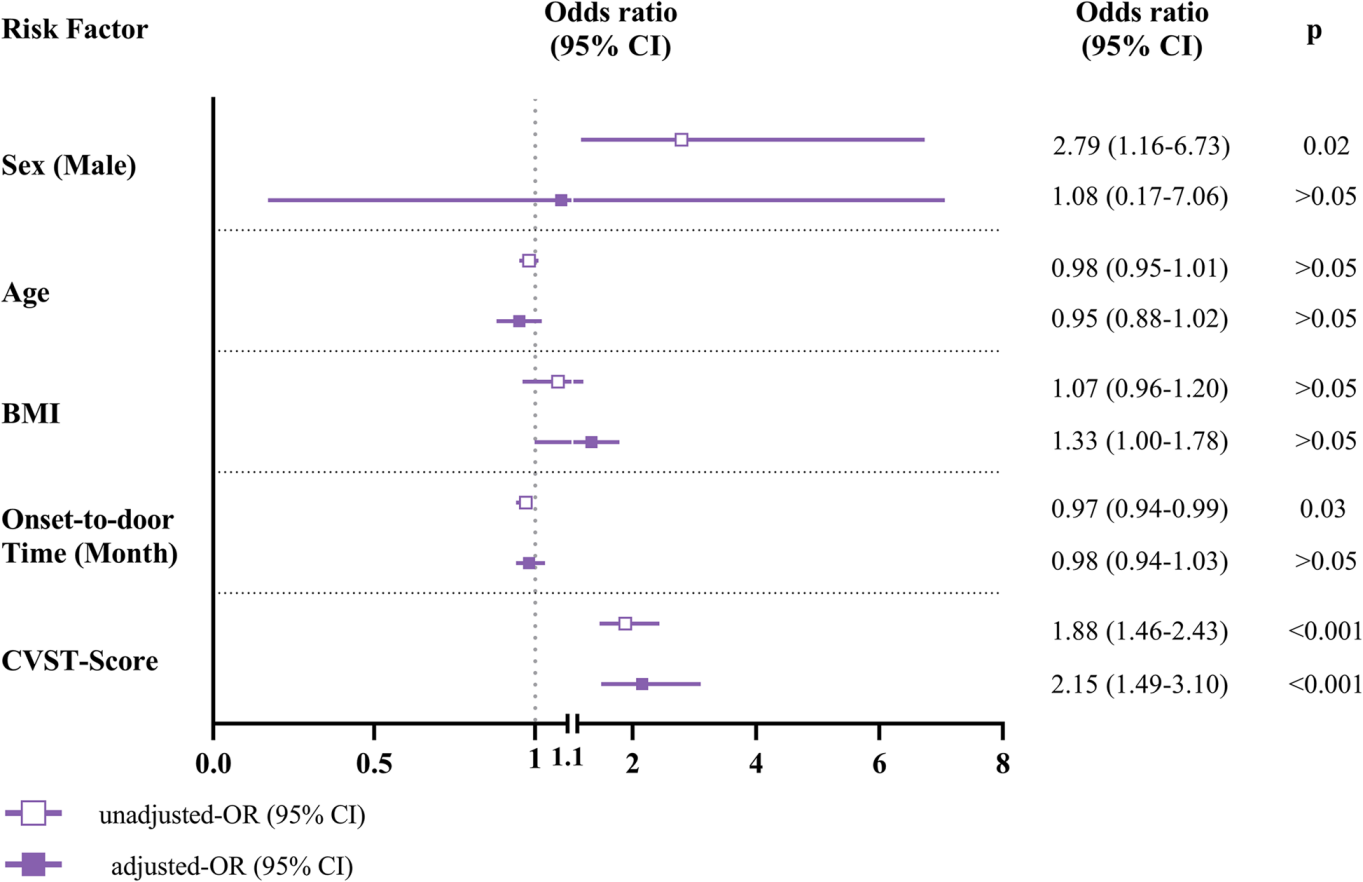
Forest plot of the risk factors for predicting ICP≥250mmH_2_O. Forest plot of univariable and multivariable logistic regression models for predicting ICP≥250mmH_2_O with the five variables including CVST-Score, sex, onset-to-door time, age, and BMI. Data are displayed using ORs and 95% CIs. Abbreviations: CVST, cerebral venous sinus thrombosis; BMI, body mass index; ICP, intracranial pressure; OR, odds ratio; CI, confidence interval

Finally, we analyzed the ability of the novel CVST-Score to predict ICP≥250mmH_2_O with logistic regression models by adjusting for potential confounding factors. In the univariate logistic regression analysis, sex, onset-to-door time, and CVST-Score were associated with ICP≥250 mmH_2_O (p = 0.02, p = 0.03 and *p* < 0.001, respectively). In the multivariate logistic regression analysis, only the CVST-Score remained an independent predictor for ICP≥250mmH_2_O after adjusting for sex, onset-to-door time, age, and BMI (*p*< 0.001). The ORs and 95% CIs for all the variables are detailed in Fig. 5. Furthermore, considering the possible impact of cerebral infarction on ICP and the sample size in this study, a second multivariate logistic regression model was set up with five other variables including, sex, onset-to-door time, CVST-Score, BMI, and cerebral infarction. Intriguingly, CVST-Score was still the only independent predictor for ICP≥250mmH_2_O (*p*< 0.001), details in Fig. S3.

## Discussion

In this study, we proposed a novel CVST-Score based on the MRBTI sequence to estimate the thrombus burden semi-quantitatively, which was closely related to the ICP, revealing its potential to predict intracranial hypertension noninvasively. We found that CVST-Score in the high ICP subgroup was significantly higher than that in the normal ICP subgroup, indicating a potential correlation between CVST-Score and ICP. The result was further verified with univariate analysis; in patients with ICP ≤330mmH_2_O, the CVST-Score was linearly and positively correlated with ICP. Therefore, the CVST-Score can potentially monitor ICP noninvasively during regular follow-up visits. Furthermore, we investigated the diagnostic capacity of the CVST-Score by plotting the ROC curves at ICP cut-off values of 250 mmH_2_O and 330 mmH_2_O, commonly used for ICP grading. We found that the CVST-Score showed both good sensitivity and specificity, suggesting that the CVST-Score can be used to predict intracranial hypertension initially. Due to differences in demographics and imaging findings among the ICP subgroups, logistic regression models were applied to explore potential factors of intracranial hypertension. A total of five variables were simultaneously entered into the first equation: sex, age, BMI, onset-to-door time, and CVST-Score. Only CVST-Score remained an independent predictor of ICP≥250mmH_2_O. Moreover, in the second multivariate logistic regression model, when we entered cerebral infarction into the equation, CVST-Score was still the only independent predictor of ICP≥250mmH_2_O. The results indicated that the primary cause of intracranial hypertension in CVST was most likely due to increased thrombus burden rather than due to other factors, which further confirms that the CVST-Score is highly predictive of ICP.

The novel CVST-Score had several advantages compared to the previous methods, which are regarded as the golden standards for reporting thrombus burden of CVST. *Zubkov et.al.* previously proposed a scoring method to record the extent and thrombus burden of CVST (the CVES method in this study) and found that the extent of CVST might be related to the incidence of brain lesions [3]. Since then, it has become a classic method to assess the thrombus volume of CVST [12, 19–22]. *Miranda and Ferro et.al.* proposed a similar score, CVOS, to estimate the thrombus burden and recanalization of CVST based on whether the venous sinus was fully occluded or not; this method has been commonly used in recent clinical research studies [1, 11, 17, 18, 23]*. Aguiar et.al.* further applied the CVOS method and found that the venous recanalization was associated with regression of brain lesions [1]. However, these methods only roughly described the location and number of the involved sinuses using a binary scoring rule at each segment. As a result, these methods could not discriminate the thrombus burden at each segment accurately or reflect the dominant transverse sinus being critical for ICP [24]. When comparing the diagnostic capacities among these methods, only the CVST-Score showed adequate, balanced sensitivity and specificity in predicting intracranial hypertension. Since the CVES could not discriminate between full and partial occlusion, this may be responsible for its low specificity, especially for diagnosing the ICP>330 mmH_2_O. Similarly, the CVOS considered partial occlusion and no thrombosis to be the same degree, likely causing its low sensitivity in diagnosing intracranial hypertension. Moreover, the two conventional methods could only record an integer number with a limited measuring range. As shown in ROC analysis, the optimal cut-off points for ICP≥250 mmH_2_O and ICP>330 mmH_2_O were nearly equal to each other, which further illustrates the low resolution of the two conventional methods at discriminating different thrombus burdens and diagnosing different ICP levels. The deficiencies of their scoring methods may be attributed to the limited imaging techniques available at that time [25, 26].

Compared to previous methods, the novel CVST-Score is the first to divide the unilateral transverse sinus and sigmoid sinus into two segments, corresponding to their long route and complex collateral branches at the skull base [27, 28]. In addition, the transverse sinus has been reported to be asymmetric in approximately 80% of the population. Therefore, whether the involved sinus is on the dominant side is critical for determining the ICP level in CVST [24, 29]. Due to the asymmetry of the transverse sinuses, the weighted drainage of bilateral transverse sinuses was calculated according to the maximal cross-sectional area. Moreover, the sum of bilateral weighted scores was equal to the total score at midline venous sinuses, which displayed the bilateral shunting function for the superior sagittal sinus, straight sinus and torcular herophili. Additionally, the thrombus burden at each segment was distinguished by the filling volume using the advantageous nature of MRBTI to visualize the thrombus and vessel wall directly [14]. As such, this novel CVST-Score could record thrombus burden at different segments semi-quantitatively to dynamically evaluate the therapeutic effects. More importantly, the scoring method did not require the cumbersome and time-consuming post-imaging analysis that would render it impractical in the urgent clinical setting.

The compensatory response of cerebral venous system may not be strong enough in patients with CVST. We previously believed that rich collateral networks would be formed with increasing disease duration, and the increased onset-to-door time seemed to reduce the risk of intracranial hypertension in the univariate logistic regression in this study. However, the correlation was insignificant after adjusting for potential confounders, indicating that multiple factors may influence the relationship between onset-to-door time and ICP. Therefore, the collateral vessels may not be adequate to compensate for the obstructed cerebral venous outflow, even with increasing disease duration. Previous studies have shown that collateral vessels in CVSS were less prevalent than those in internal jugular venous stenosis, contributing to the higher ICP of CVSS. In addition, venous collaterals were not independently related to clinical manifestations and outcomes of CVST, which also reflects the inadequate compensation of the recruited collateral vessels [30, 31].

The novel CVST-Score has good utility for clinical applications. For example, it can be used to help stratify if patients need to undergo invasive procedures, such as lumbar puncture or endovascular treatment for CVST, which are not typically the routine standard of care [9]. For patients who are predicted to be at high risk of intracranial hypertension at admission from the CVST-Score, a lumbar puncture can be applied to confirm the ICP, and endovascular treatment could be performed urgently for early recanalization [9, 13]. On the other hand, for patients whom the CVST-Score predicates to be at low risk for developing intracranial hypertension, medical treatment with therapies such as anticoagulants and acetazolamide would be the preferred initial treatment [9], following which the CVST-Score can be used as an indicator to assess therapeutic responses. Moreover, the ICP≥330mmH_2_O has been proven to predict the deterioration of visual impairment [32]. The CVST-Score had good sensitivity and specificity in predicting ICP≥330mmH_2_O at the cut-off point of 11.62. For patients at high risk of ICP≥330mmH_2_O, more invasive procedures, such as optic nerve sheath fenestration, could be considered to avoid irreversible visual damage when recanalization cannot be achieved [9, 32]. Additionally, we also believe that the CVST-Score can be used in the outpatient setting to determine if patients need lumbar puncture. Currently, there is a lack of consensus on whether patients should undergo routine lumbar puncture in the outpatient setting because patients are commonly on oral anticoagulation, predisposing them to an increased risk of hemorrhage [13]. The CVST-Score could screen patients at high risk of intracranial hypertension to undergo lumbar puncture while helping patients at low risk of intracranial hypertension avoid unnecessary invasive procedures.

For patients with incomplete recanalization in the post-acute phase of CVST, their nonspecific residual symptoms were often overlooked during regular follow-up, partly due to the lack of dedicated scoring methods to guide clinical practice. *Hiltunen et.al.* found that 68% of patients with CVST had residual symptoms such as neuropsychological problems, linguistic difficulties, and headaches, hindering their ability to return to previous work [6]. *Ji et.al.* found that severe headache was an important residual symptom in CVST, which was independently correlated with isolated intracranial hypertension and failed recanalization, and may contribute to the unfavorable outcome [7]. The available methods to evaluate the outcome of CVST, such as mRS, could not discriminate these residual symptoms adequately [33, 34]. Consequently, we should focus on the patient’s complaints comprehensively to recognize the specific sequelae during long-term follow-up and set up specialized evaluation methods for CVST [34]. In our study, headaches and visual impairment were still the most common symptoms despite the varying span of onset-to-door time. The thrombus burden was independently correlated with ICP by the CVST-Score. ICP is a significant contributor to headaches and visual impairment in CVST. Due to the noninvasive advantage of MRBTI, we believe that the CVST-Score shows promise as a specialized method for evaluating the severity and therapeutic responses of CVST during long-term follow-up.

There are several limitations to our study. Firstly, most CVST patients were at a subacute or chronic stage in our study. Due to emergent endovascular treatment in our center, acute CVST was rarely included in our study because of insufficient imaging data. However, the influence of onset-to-door time on ICP may not be significant because it is not an independent risk factor of intracranial hypertension. Nevertheless, the ability of CVST-Score to predict ICP in the acute stage requires further validation. Secondly, the linear relationship between CVST-Score and ICP was not settled at ICP >330mmH_2_O because the measuring range of the manometer was from 50mmH_2_O to 330mmH_2_O. The lumbar puncture kit in our hospital did not have an extension tube to measure higher ranges. However, an increasing trend of CVST-Score from ICP <250mmH_2_O to >330mmH_2_O might imply the potential predictive utility. Additionally, the collateral vessels were not involved in calculating the CVST-Score because MRBTI suppressed the blood signal. However, the onset-to-door time was unrelated to ICP after adjustment, indicating that the collateral vessels formed during the recovery period may not be adequate to compensate for the increased ICP. Moreover, the thrombus burden at each segment may partly reflect the potential of collateral formation by the occluded openings and increased flow resistance. Further prospective multicenter studies of larger sample sizes are required to validate the accuracy of CVST-Score and evaluate the combined effect of thrombus burden and collateral circulation with other imaging methods.

## Conclusions

Our study proposes a novel and patient-specific CVST-Score based on MRBTI to estimate the thrombus burden of CVST comprehensively without the cumbersome and time-consuming post-imaging analysis. The novel score showed better diagnostic performance for intracranial hypertension than other conventional methods. Moreover, the CVST-Score was closely and positively correlated with ICP after adjusting for confounders, indicating its potential to predict intracranial hypertension noninvasively. As such, the CVST-Score can be leveraged to create a specialized method to evaluate the severity and therapeutic responses of CVST and can be used to avoid unnecessary invasive procedures at long-term follow-up.

## Supplementary Information

**Additional file 1: Fig. S1.** The CVES in ICP subgroups and diagnostic accuracy of the CVES.

**Additional file 2: Fig. S2.** The CVOS in ICP subgroups and diagnostic accuracy of the CVOS.

**Additional file 3: Fig. S3.** Forest plot of the risk factors for predicting ICP≥250mmH_2_O with cerebral infarction included. Forest plot of univariable and multivariable logistic regression models for predicting ICP≥250mmH_2_O with the five variables including CVST-Score, sex, onset-to-door time, BMI, and cerebral infarction. Data are displayed using ORs and 95% CIs. Abbreviations: CVST, cerebral venous sinus thrombosis; BMI, body mass index; ICP, intracranial pressure; OR, odds ratio; CI, confidence interval.

**Additional file 4: Table S1.** A designed record form to calculate CVST-Score.

**Additional file 5: Table S2.** The diagnostic capacities of the three different scoring methods for ICP

## Data Availability

The datasets generated and/or analysed during the current study will be made available from the corresponding author on reasonable request.

## Abbreviations

CVST: Cerebral venous sinus thrombosis
CE-MRV: Contrast-enhanced magnetic resonance venography
CTV: Computed tomography venography
ICP: Intracranial pressure
MRBTI: Magnetic resonance black-blood thrombus imaging
mRS: Modified rankin scale
CVES: Cerebral venous extent score
CVOS: Cerebral venous occlusion score
BMI: Body mass index
CVSS: Cerebral venous sinus stenosis
CSF: Cerebrospinal fluid
