# Supplementary figures and images for: A novel score to estimate thrombus burden and predict intracranial hypertension in cerebral venous sinus thrombosis
Source: J Headache Pain. 2023 Mar 17;24(1):29. doi: 10.1186/s10194-023-01562-9 (PMC10022088; doi:10.1186/s10194-023-01562-9)

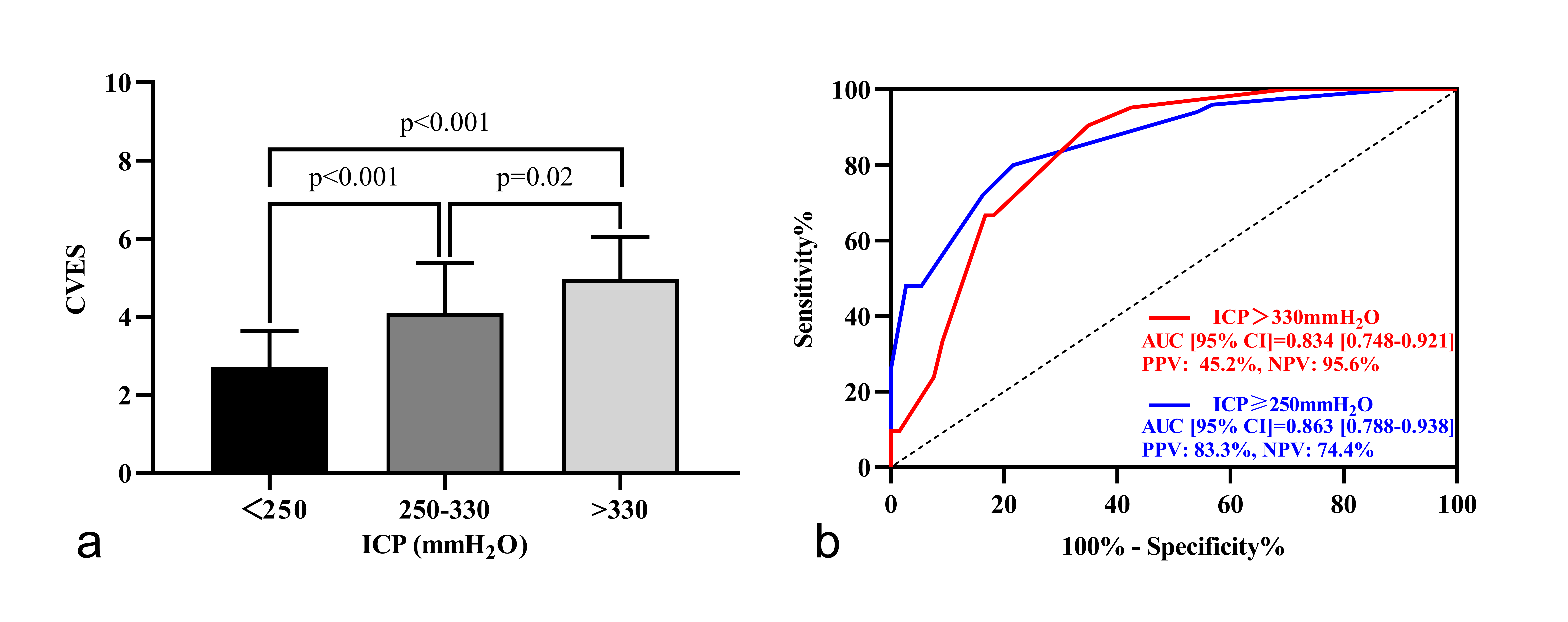

Supplement: Supplementary file 1 — Additional file 1: Fig. S1. The CVES in ICP subgroups and diagnostic accuracy of the CVES. [file 10194_2023_1562_MOESM1_ESM.tif]

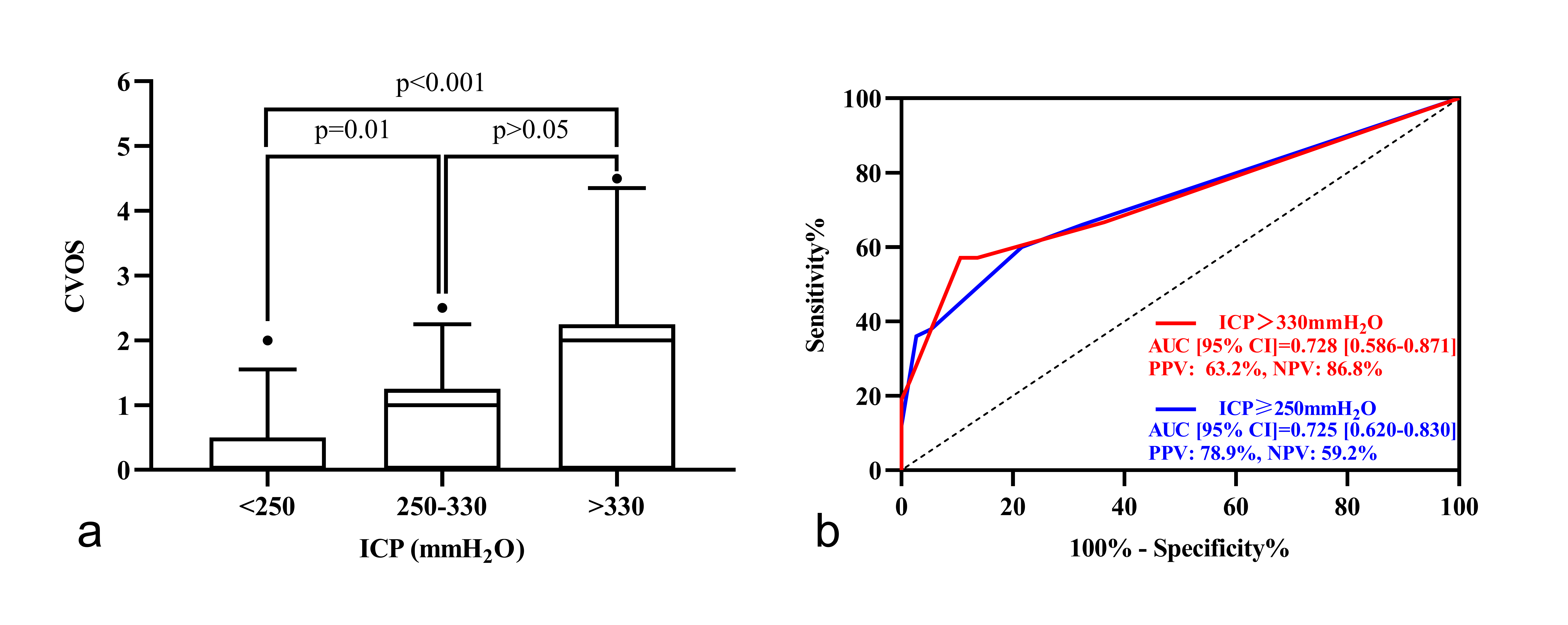

Supplement: Supplementary file 2 — Additional file 2: Fig. S2. The CVOS in ICP subgroups and diagnostic accuracy of the CVOS. [file 10194_2023_1562_MOESM2_ESM.tif]

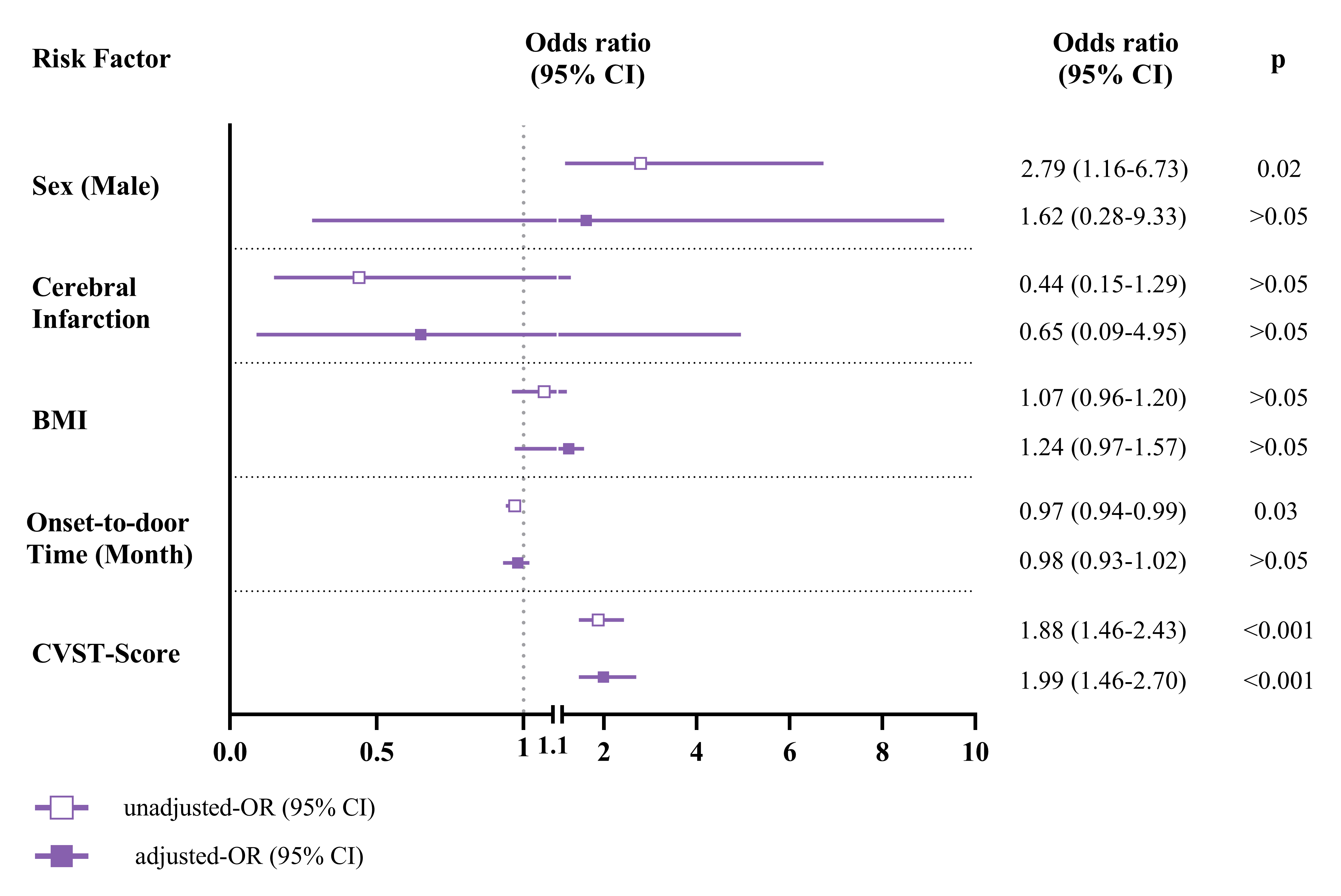

Supplement: Supplementary file 3 — Additional file 3: Fig. S3. Forest plot of the risk factors for predicting ICP≥250mmH2O with cerebral infarction included. Forest plot of univariable and multivariable logistic regression models for predicting ICP≥250mmH2O with the five variables including CVST-Score, sex, onset-to-door time, BMI, and cerebral infarction. Data are displayed using ORs and 95% CIs. Abbreviations: CVST, cerebral venous sinus thrombosis; BMI, body mass index; ICP, intracranial pressure; OR, odds ratio; CI, confidence interval. [file 10194_2023_1562_MOESM3_ESM.tif]
