# Supplementary material for: A novel score to estimate thrombus burden and predict intracranial hypertension in cerebral venous sinus thrombosis
Source: J Headache Pain. 2023 Mar 17;24(1):29. doi: 10.1186/s10194-023-01562-9 (PMC10022088; doi:10.1186/s10194-023-01562-9)
Supplement: Supplementary file 4 — Additional file 4: Table S1. A designed record form to calculate CVST-Score. [file 10194_2023_1562_MOESM4_ESM.docx]

**Table S1.** A designed record form to calculate CVST-Score

| STORIJ (CVST-Score) | | | | | | | | | |
| --- | --- | --- | --- | --- | --- | --- | --- | --- | --- |
| Midline | S_1_ | S_2_ | S_3_ | T | O | SUM_M_ | CVST-Score | | |
|  |  |  |  |  |  |  |  | | |
| Left | R_1_ | R_2_ | I_1_ | I_2_ | J_1_ | SUM_L_ | CS_L_ | W_L_ |  |
|  |  |  |  |  |  |  |  |  |  |
| Right | R_3_ | R_4_ | I_3_ | I_4_ | J_2_ | SUM_R_ | CS_R_ | W_R_ |  |
|  |  |  |  |  |  |  |  |  |  |
| Abbreviations:  Firstly, S_1_, S_2_ and S_3_ represented the anterior one-third, the intermediate one-third, and the posterior one-third of the superior sagittal sinus, respectively; T: straight sinus; O: torcular herophili; SUM_M_ was the sum score of these aforementioned midline sinuses.  Secondly, R_1_ and R_2_ represented the medial half and the lateral half of the left transverse sinus; I_1_ and I_2_ represented the posterior segment and anterior segment of the left sigmoid sinus (namely, the vertical segment and horizontal segment); J_1_: left internal jugular vein; SUM_L_ was the sum score on the left side of cerebral venous sinuses.  Correspondingly, R_3_ and R_4_ represented the medial half and the lateral half of the right transverse sinus; I_3_ and I_4_ represented the posterior segment and anterior segment of the right sigmoid sinus (namely, the vertical segment and horizontal segment); J_2_: right internal jugular vein; SUM_R_ was the sum score on the right side of cerebral venous sinuses.  Lastly, CS_L_: the maximal cross-sectional area of left transverse sinus; CS_R_: the maximal cross-sectional area of right transverse sinus; W_L_: the weighted drainage of left transverse sinus; W_R_: the weighted drainage of right transverse sinus; CVST-Score: the total thrombus score by summing up the SUM_M_, the weighted SUM_L_ and the weighted SUM_R_. | | | | | | | | | |
