# Supplementary material for: A novel score to estimate thrombus burden and predict intracranial hypertension in cerebral venous sinus thrombosis
Source: J Headache Pain. 2023 Mar 17;24(1):29. doi: 10.1186/s10194-023-01562-9 (PMC10022088; doi:10.1186/s10194-023-01562-9)
Supplement: Supplementary file 5 — Additional file 5: Table S2. The diagnostic capacities of the three different scoring methods for ICP [file 10194_2023_1562_MOESM5_ESM.docx]

**Table S2.** The diagnostic capacities of the three different scoring methods for ICP

|  | **ICP (mmH_2_O)** | **AUC** | **95%CI** | **Sensitivity** | **Specificity** | **PPV** | **NPV** |
| --- | --- | --- | --- | --- | --- | --- | --- |
| CVST-Score | ≥250 | 0.956 | 0.919-0.994 | 94.0% | 86.5% | 90.4% | 91.4% |
|  | ＞330 | 0.895 | 0.830-0.961 | 90.5% | 80.3% | 59.4% | 96.4% |
| CVES | ≥250 | 0.863 | 0.788-0.938 | 80.0% | 78.4% | 83.3% | 74.4% |
|  | ＞330 | 0.834 | 0.748-0.921 | 90.5% | 65.2% | 45.2% | 95.6% |
| CVOS | ≥250 | 0.725 | 0.620-0.830 | 60.0% | 78.4% | 78.9% | 59.2% |
|  | ＞330 | 0.728 | 0.586-0.871 | 57.1% | 89.4% | 63.2% | 86.8% |

Abbreviations: CVST-Score, the proposed novel scoring method for thrombus burden of cerebral venous sinus thrombosis in this study; CVES, cerebral venous extent score; CVOS, cerebral venous occlusion score; ICP, intracranial pressure; AUC, area under the curve; CI, confidence interval; PPV, positive predictive value; NPV, negative predictive value.
